# Permanent occlusion of the Eustachian tube: a retrospective study on reopening procedures

**DOI:** 10.1007/s00405-023-08271-8

**Published:** 2023-10-17

**Authors:** Holger Sudhoff

**Affiliations:** Department of Otorhinolaryngology, Head and Neck Surgery, Medical Faculty OWL, Bielefeld University, Campus Klinikum Bielefeld, Teutoburger Str. 50, 33604 Bielefeld, Germany

**Keywords:** Permanent occlusion of the Eustachian tube, Eustachian tube reopening, Dilatory Eustachian tube dysfunction, Middle ear effusion, Glue ear, Glue Eustachian tube

## Abstract

**Purpose:**

This study retrospectively evaluated the efficacy and versatility of reopening procedures for the permanent occlusion of the cartilaginous Eustachian tube (POET) by analyzing four consecutive cases.

**Methods:**

The study included all patients diagnosed with POET who suffered from Eustachian tube occlusion and glue ear. A combined approach of endoscopic transnasal/transoral laser surgery was utilized to reopen the POET. This was subsequently followed by balloon dilation (BET) and stenting for a duration of six weeks. In one distinct case, the Eustachian tube orifice was approached via a transtympanic method, where a balloon catheter was placed. The primary outcome measures targeted the success rate of reopening, which was quantified using audiological outcomes and Eustachian tube patency verified by a positive Valsalva maneuver.

**Results:**

Four patients, with an age range of 14–62 years (mean age of 29.3 years), were subject to Eustachian tube reopening. The duration of follow-up varied between 10 and 24 months, averaging at 16.2 months. Notably, 75% of the surgically treated ears displayed no evidence of glue ear upon their last follow-up and showed restoration of Eustachian tube patency. The procedures were executed without any surgical complications. The causes for POET in these patients were heterogeneous: two were attributed to scarring post adenoidectomy, one to occlusion following orthognathic surgery and the remaining one due to prior radiotherapy treatment for squamous cell carcinoma located at the soft palate.

**Discussion:**

Total occlusion of the cartilaginous Eustachian tube may be linked to persistent middle ear diseases. It is imperative to conduct nasopharyngeal endoscopy in these cases. The findings from this study suggest that the Eustachian tube reopening procedure is predominantly effective and safe for patients with POET stemming from a variety of pathologies. Future research should focus on exploring advanced stenting devices and necessitate longer follow-up periods for comprehensive understanding.

## Introduction

Eustachian tube dysfunction (ETD) and permanent occlusion of the Eustachian tube (POET) are two otological entities with significant clinical implications. While both share a common anatomical region, their etiologies, manifestations, and treatments differ considerably. Chronic middle ear diseases often associate with POET, resulting from compromised pressure equalization and drainage capacities [1]. On the other hand, ETD encompasses a broader spectrum of underlying pathologies.

Distinguishing POET from functional Eustachian tube (ET) obstruction or dilatory ET dysfunction is crucial [2, 3]. Various mechanisms can cause POET. This includes complications from surgical procedures, radiotherapy, and numerous rheumatological disorders that induce chronic inflammation or mechanical obstructions [4, 5]. Surgical interventions, particularly adenoidectomy, orthognathic surgery, sinus surgery, and turbinectomy, often result in scar formation or a distorted nasopharyngeal anatomy. This culminates in a non-functional ET, causing conditions like glue ear, chronic otitis media, and cholesteatoma [6–8].

ETD is multifaceted and often stems from a functional mucosal obstruction within the cartilaginous portion of the ET [9]. This impairs the ventilation of the middle ear and mastoid cavity, resulting in conditions like glue ear and chronic otitis media. The subsequent diseases may manifest as tympanic membrane perforations, ossicular chain erosion, and cholesteatoma. Consequently, patients may experience impaired hearing thresholds or even deafness [10–12]. Those with POET typically present with aural fullness, conductive hearing loss, and often undergo procedures such as myringotomy, grommets, or middle ear surgeries.

Lesions of the nasopharynx, radiotherapy of the adjacent tissue, and surgical interventions around the ET have a potential risk of otitis media with effusion [13, 14].

Several factors can cause or exacerbate ETD and POET. Lesions in the nasopharynx, adjacent tissue radiotherapy, and surgical interventions around the ET can precipitate otitis media with effusion [13, 14]. However, achieving a permanent Eustachian tube occlusion during translabyrinthine acoustic neuroma surgeries often meets limited success [15]. Other systemic diseases, like sarcoidosis, tuberculosis, and granulomatosis with polyangiitis, can lead to inflammation and subsequent closure of the pharyngeal ET orifice, manifesting as crusting, swelling, and scarring [16]. While dilatory ET dysfunction (DETD) rarely results in complete ET lumen occlusion, it does allow for balloon dilation Eustachian tuboplasty [9]. Predominantly, in POET patients, the nasopharyngeal orifice or the cartilaginous portion is affected. Conservative treatment or balloon dilation tuboplasty alone cannot restore this condition. This particular entity significantly impacts the patient’s quality of life due to the associated hearing impairment and other otological symptoms [17, 18].

Given the complexities surrounding ETD and POET, this preliminary study focuses on exploring the potential of surgically reopening the ET for patients diagnosed with POET. By addressing this lesser-known otological disorder, this research aims to shed light on its implications and therapeutic interventions, raising awareness, and contributing to improved clinical outcomes.

## Materials and methods

This retrospective analysis was carried out in a University medical center. The study comprised four consecutive patients who were diagnosed with long-standing permanent occlusion of the Eustachian tube (POET) specifically at the nasopharyngeal orifice or within the cartilaginous portion of the ET. The primary clinical manifestation in all patients was intractable glue ear, a condition known for its recurrent and persistent middle ear effusion.

### Patient cohort

Upon initial presentation, these patients were subjected to the insertion of tympanostomy tubes as an attempt to manage their glue ear condition. Subsequent developments in two of these patients led to cholesteatoma, a skin cyst in the middle ear, and subsequently, they underwent cholesteatoma surgery. During these interventions, the presence of POET as a potential underlying cause was overlooked.

After a comprehensive clinical evaluation and upon realizing the chronicity and persistence of the symptoms, all four patients were counseled and then consented for Eustachian tube reopening surgery. This surgical procedure not only aimed at reopening the occluded Eustachian tube but also included the placement of stents to maintain the patency postoperatively. Additionally, ET probing was conducted via the middle ear to further evaluate the integrity and functionality of the Eustachian tube.

1. Patient reporting: Patients were routinely asked about the symptoms of Eustachian tube dysfunction to track any recurrence or alleviation of the symptoms.
2. Otomicroscopy: To visualize and examine the middle ear's structures.
3. Flexible fiberoptic nasopharyngoscopy: Employed to inspect the nasopharynx and the posterior aspect of the Eustachian tube.
4. Tympanometry: To measure the movement of the tympanic membrane in response to changes in air pressure, reflecting the middle ear's pressure.
5. Pure tone audiometry: A hearing test to measure both air and bone conduction hearing thresholds, ensuring no residual hearing impairment postoperatively.
6. Tubomanometry: Specifically used to evaluate the functional status of the Eustachian tube by measuring its pressure and patency.

Through this comprehensive approach, the study aimed to present a holistic view of the efficacy of Eustachian tube reopening surgery in patients with long-standing POET, contributing substantially to the current otological literature.

Postoperative procedure and follow-up for Eustachian tube reopening.

Ensuring the surgical success and vigilantly monitoring any complications were paramount. The postoperative assessments were carefully designed to encapsulate the entire surgical outcome.

### Operative technique

**Fig. 1:**
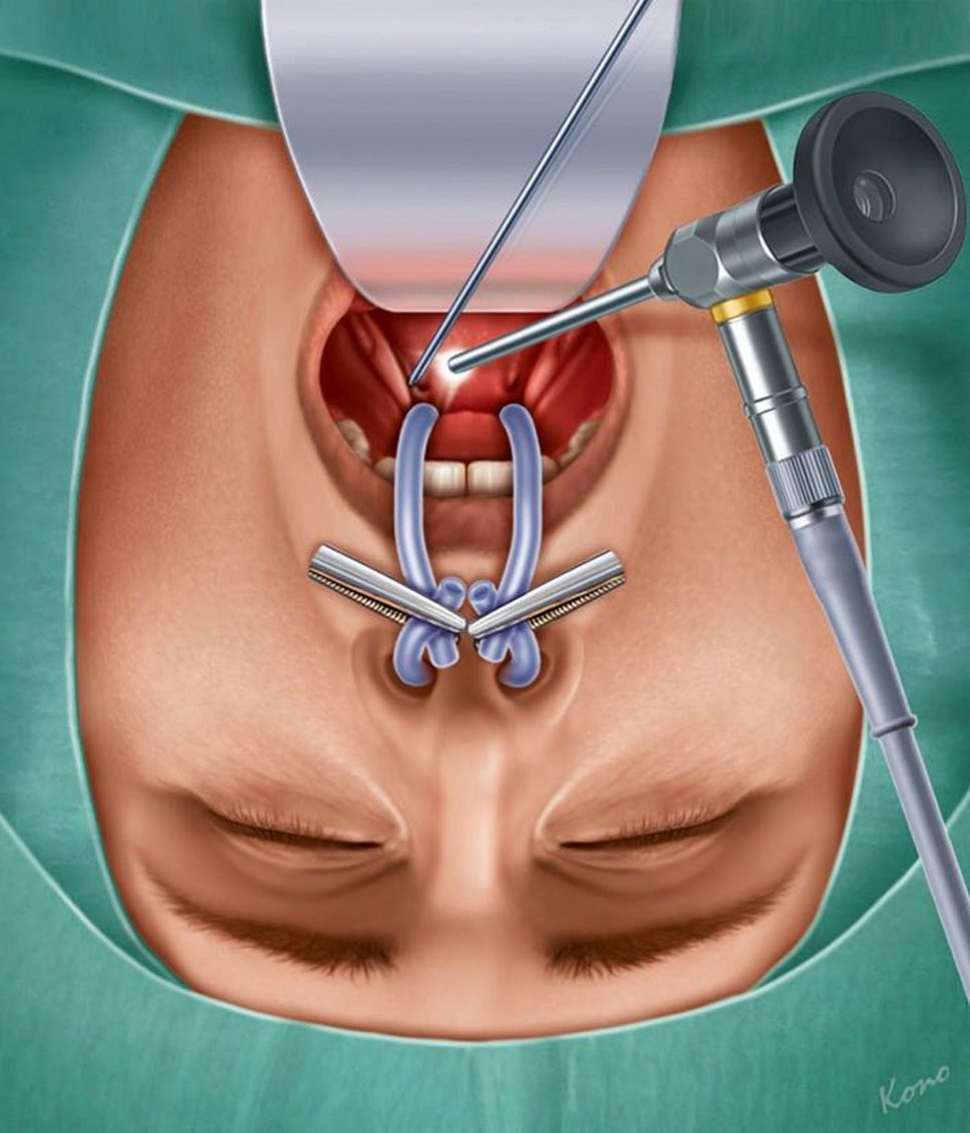
After insertion of a mouth gag, velotraction with suction catheters was established. Transoral endoscopy was performed with a rigid 70° Hopkins optic and the 445 nm blue laser

**Fig. 2:**
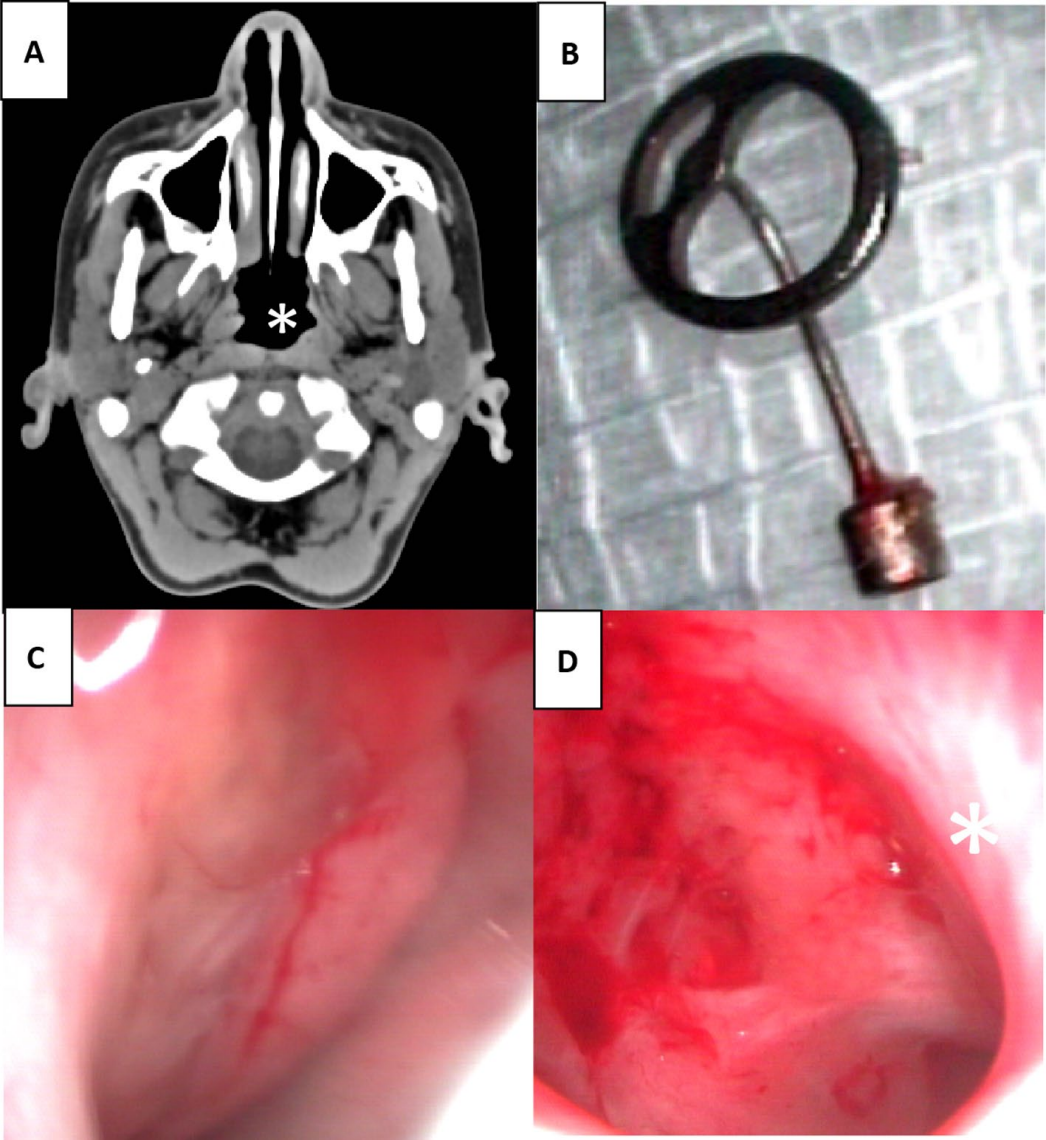
**A** Axial view of temporal CT showed complete deformity of the left Eustachian tube orifice (*). A 14-year-old male Caucasian patient underwent adenoidectomy at the age of 1 year and revision adenoidectomy at the age of 2. At the age of 3 years, he developed a left-sided cholesteatoma requiring 9 prior revision surgeries. **B** The left middle ear total ossicular chain prosthesis was severely bent due to the long-term negative pressure caused by POET. **C** and **D** The right orifice was not as severely damaged as the left orifice (*). This case required revision surgery for after reocclusion

**Fig. 3:**
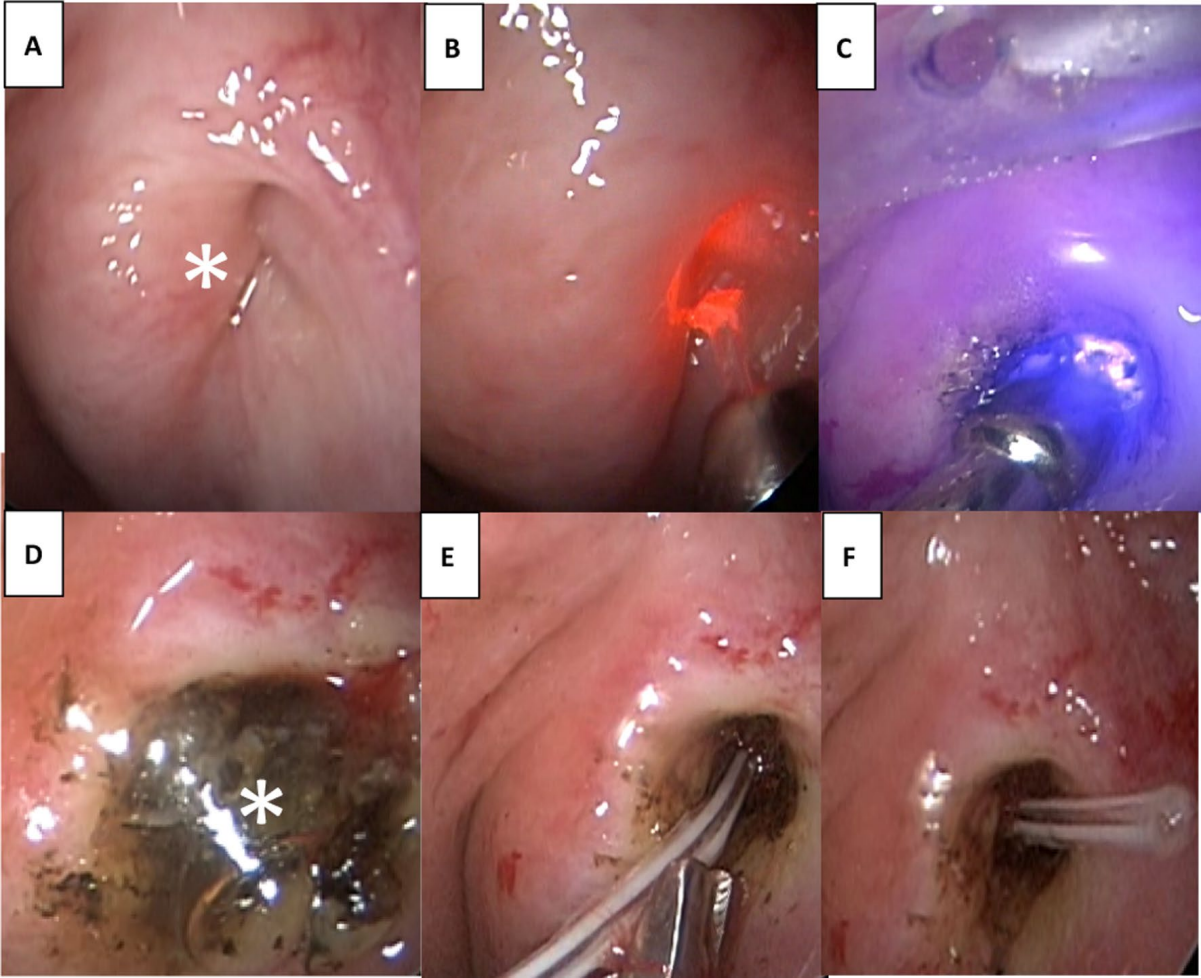
Endoscopic images of a 62-year-old female Caucasian patient after orthognathic surgery (Maxillomandibular advancement surgery) right ET. **A** POET of the lumen across the entire nasopharyngeal orifice. **B** Application of the laser to the occluded orifice. **C** Parotid duct probe visible through obliterated ET orifice. **D** Release of glue through the re-opended ET (*). **D** Angiocatheter stent inserted and **E** introduced in full length of ET

The procedure began with velotraction, employing a rigid 70° Hopkins endoscope for transoral visualization beneath the soft palate (Fig. 1). With the aid of a tonsillectomy mouth gag and velotraction using suction catheters, surgeons obtained a clear view of the surgical field. The anatomical symmetry of the two pharyngeal ET orifices acted as a crucial guide (Fig. 2). Surgeons meticulously inspected the ET orifice and gently palpated with blunt instruments to discern any manipulative movements from the parotid duct probe through the occlusion. A 445 nm blue laser (TruBlue^®^, A.R.C. Laser Company) was used. Positioned into a bent suction device, it was directed toward the affected pharyngeal ET orifice (Fig. 2). Crucial landmarks, including remnants of the torus tubarius and its medial cartilaginous lamina, were identified for guidance. The laser was systematically used to expose the parotid duct probe, post which the lumen was widened further (Fig. 3).

An evident sign of progress was the release of mucus from the Eustachian tube. Surgeons meticulously continued dissection until they could identify the mucosa-lined ET lumen. A standard BET was performed on the cartilaginous portion of the ET, applying 10 bars of pressure for a duration of 2 min (Device: Siggle&Theis, Overath, Germany). Post dilation, an angiocatheter stent was transorally positioned into the newly formed ET lumen. This was firmly secured using non-resorbable stitches, ensuring its location just inside the anterior pillar. A crucial step was to inspect that the catheter remained invisible through the myringotomy within the middle ear. This stent remained in place for a duration of 6 weeks.

**Fig. 4:**
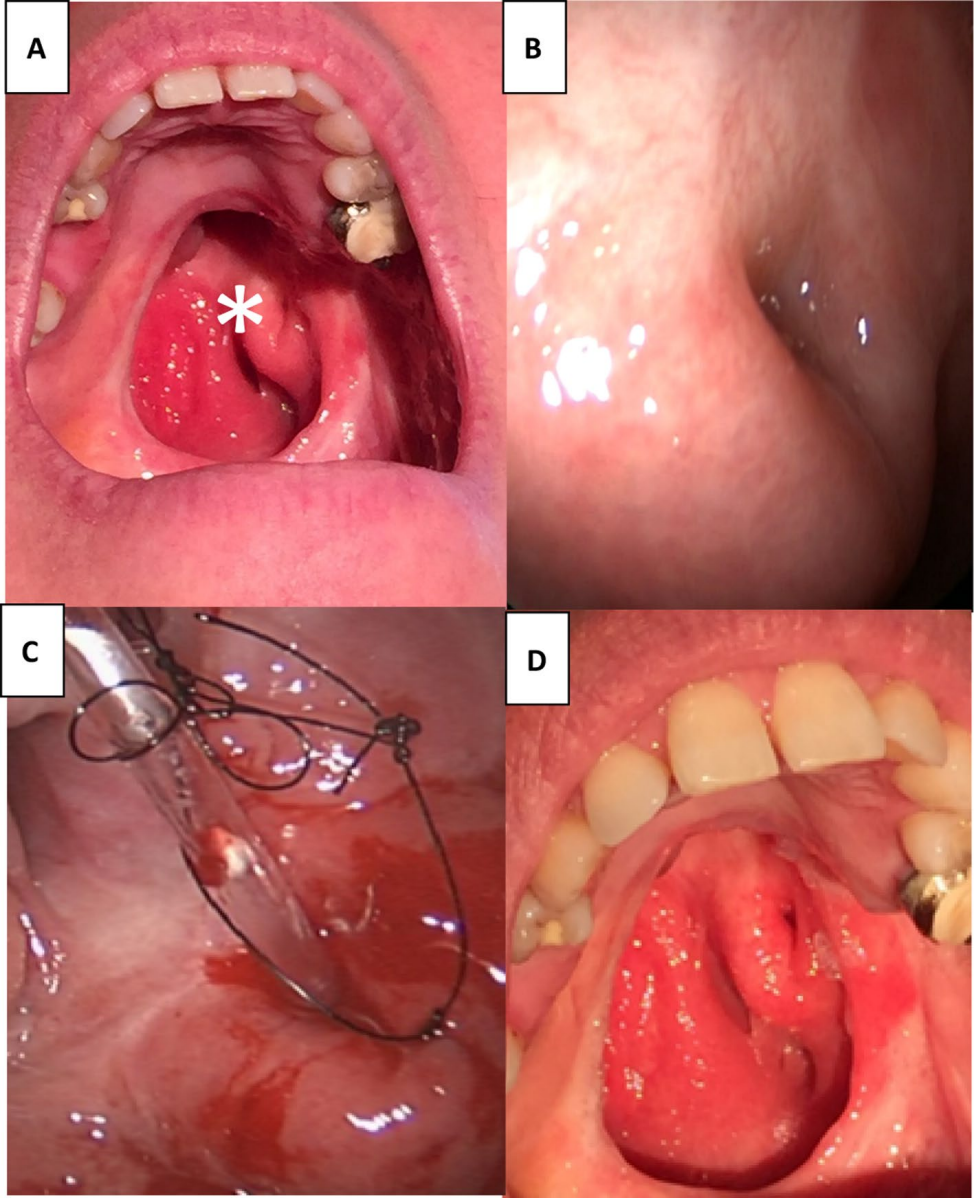
**A** Endoscopic images of a 46-year-old female Caucasian patient after previous radiotherapy for a squamous cell carcinoma of the soft palate occluded with an obturator and left POET (**B**). **C** The removal of the obturator allowed a direct approach via the soft palate to secure stenting. **D** Reopened lumen with a patent ET and regular middle ear function 8 months after primary ET surgery

**Fig. 5:**
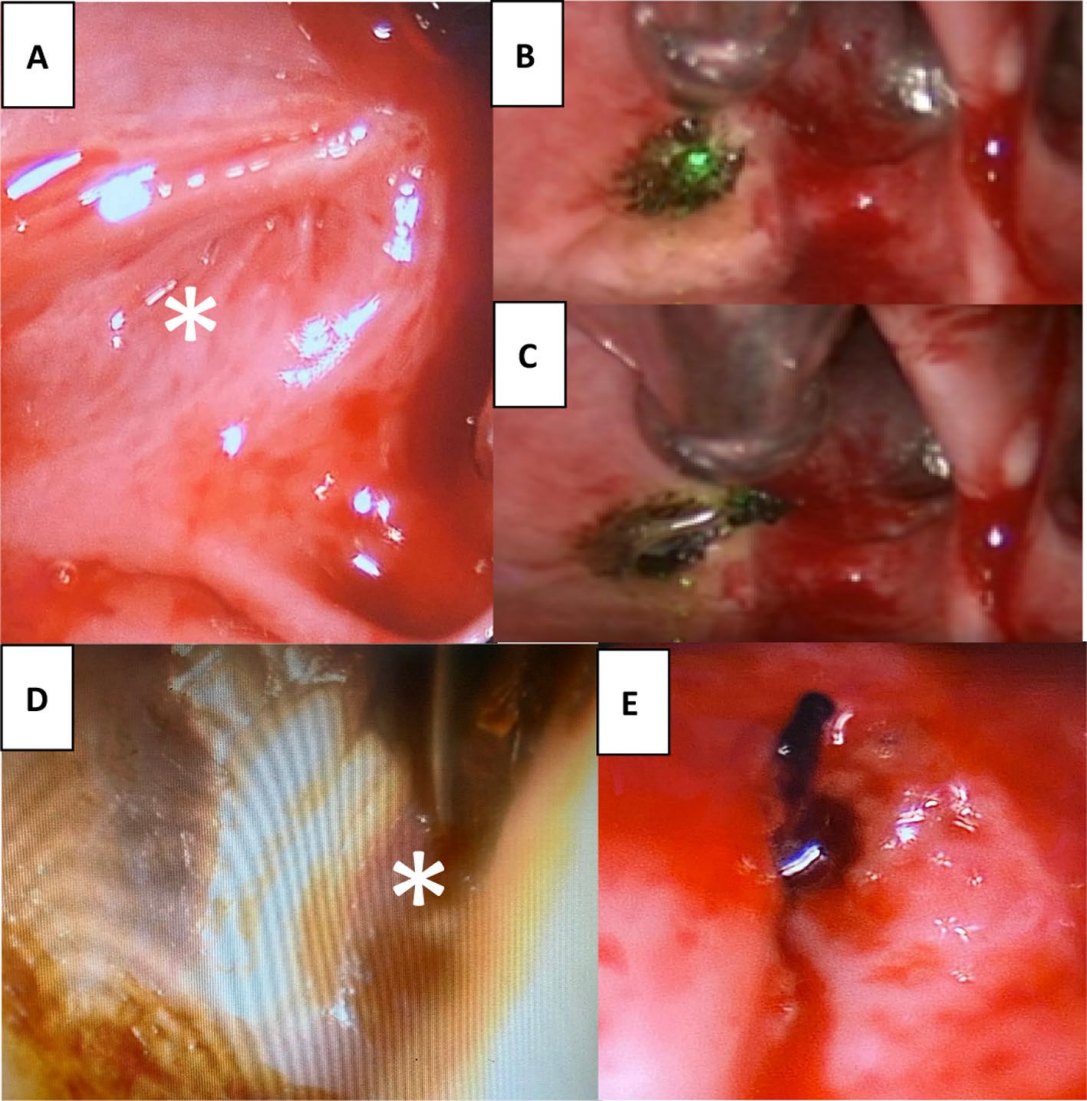
Endoscopic images of patient 4, a 28-year-old female Caucasian patient. She underwent adenoidectomy at the 3 years of age and developed glue ear with repeated grommets for 25 years. **A** POET of the lumen across the entire right nasopharyngeal orifice (*). **B** Opening of the obliterated ET orifice with a laser. **C** Release of glue through the re-opended ET. **D** A balloon catheter was positioned in the nasopharyngeal orifice and secured behind the ear via the tympanic membrane after previous cartilage palisades tympanoplasty (*). **E** The tip of the balloon catheter is positioned in the nasopharyngeal orifice and secured behind the ear for six weeks

For the patient with a history of radiotherapy for squamous cell carcinoma of the soft palate, an obturator was functionally used. Its removal permitted a direct approach via the soft palate, followed by stenting (Fig. 4). Another unique case involved patient 4, who had a balloon catheter placed through the tympanic membrane, which was positioned in the nasopharyngeal orifice. This was secured behind the ear for six weeks without complications and later removed via the ear. Post-procedure, the myringotomy healed within a week (Fig. 5). This detailed protocol offers a comprehensive insight into the operative technique, ensuring both the procedure’s safety and efficacy.

## Results

Between 2016 and 2022, a total of four patients underwent unilateral reopening of the POET. Total Number of patients was 4. Gender Distribution: Female: 3 (75%), Male: 1 (25%). Age Range: 14–62 years. Mean Age: 37.5 years. Total Number of Procedures: 5 (comprising 4 primary and 1 revision surgery). Mean Follow-up: 12.6 months. range of 14–62 years (mean age of 29.3 years), were subject to Eustachian tube reopening. The duration of follow-up varied between 10 and 24 months, averaging at 16.2 months.

All the surgical interventions were performed by the author. All patients underwent standard balloon dilation tuboplasty, a protocol to ensure the preservation of the ET lumen. The surgical procedure was mostly complication-free, emphasizing the surgical proficiency. 75% (3 out of 4 primary surgeries were successful in restoring and maintaining the ET function). Complications: Out of the four patients, one experienced a complication where scarring led to the recurrence of complete occlusion of the ET orifice. The patient with complications required a revision surgery, indicating a 25% reoperation rate.

The surgical intervention for reopening the POET demonstrated a promising outcome, with a high success rate of 75%. Only one patient experienced complications leading to revision surgery. The absence of complications in the other patients suggests the safety and efficacy of the procedure when performed by experienced professionals.

## Discussion

Patients with permanent occlusion of the cartilaginous Eustachian tube (POET) present a unique clinical challenge. In this case series, we discussed the manifestation of POET in patients and the potential interventions used to treat this rare condition. It is important to recognize that POET cases, though rare, are potentially underreported. Often, it can be an accidental discovery, overshadowed by the more prevalent Dilatory Eustachian Tube Dysfunction [9]. This further complicates its timely diagnosis. Proper diagnostic measures are pivotal. While imaging methods have their merits, endoscopy remains the gold standard to identify occlusion in the cartilaginous ET. Preoperative Valsalva CTs might offer some insights [19]. The etiology of POET is multifaceted. Surgical interventions, radiotherapy, trauma, and systemic inflammatory diseases can contribute to its occurrence [4, 5, 11, 20]. In the context of surgeries, operations such as adenoidectomy, sinus surgery, and orthognathic surgery could be potential triggers. Reopening of the ET is the mainstay treatment. However, complications like scarring can ensue, leading to reocclusion.

To mitigate this, stenting combined with balloon Eustachian tuboplasty (BET) can be employed, assisting in the healing process. Stent fixation remains a challenge, but innovative approaches, like securing it through the tympanic membrane, are being explored. One of the challenges post-surgery is the possibility of restenosis of the ET. The timing and design of stents for the Eustachian tube remain areas requiring more research and development [21, 22]. Lasers, although beneficial, might exacerbate scarring risks. With the frequency of surgeries like adenoidectomy, it is crucial to understand the possible, albeit rare, risk of developing POET. Patients manifesting specific symptoms post such surgeries should be promptly evaluated for POET. In conclusion, while POET remains a challenging condition to diagnose and manage, this case series sheds light on its complexity, offers insights into its management, and underscores the need for continued research and development in this field.

## Conclusions

These cases presented that reopening the Eustachian tube (ET) offers a potential solution for those with permanent occlusion of the cartilaginous Eustachian tube (POET). While the procedure appears safe based on the limited data, its long-term efficacy remains a question. Therefore, further research involving a larger cohort and extended post-surgical monitoring is essential. Such studies would provide more robust data, offering a clearer perspective on the success rates, potential complications, and long-term outcomes of the procedure.

Moreover, the rarity and the often-overlooked nature of POET can lead to delays in diagnosis and treatment, possibly exacerbating patient suffering and complicating treatment. It is crucial for medical professionals to be more attuned to this disorder, ensuring early identification and intervention. With the right awareness campaigns and continued research, the medical community can better serve patients experiencing this unique otological challenge.
